# Response to: Cavagna et al The importance of considering cultural and environmental elements in an interventional model of care to fight hypertension in Africa

**DOI:** 10.1111/jch.14253

**Published:** 2021-04-04

**Authors:** Harun A. Otieno, Charles Miezah, Gerald Yonga, Fred Kueffer, Molly Guy, Chemuttaai Lang’at, Douglas A. Hettrick, Roland Schmieder

**Affiliations:** ^1^ Africa Heart Associates Nairobi Kenya; ^2^ Samartex Hospital Samreboi Ghana; ^3^ School of Medicine University of Nairobi Nairobi Kenya; ^4^ Medtronic, Inc. Minneapolis MN USA; ^5^ Department of Nephrology and Hypertension University Hospital of the Friedrich‐Alexander University Erlangen‐Nürnberg Erlangen Germany

To the Editor,

We appreciate the thoughtful interest from Dr Cavagna and colleagues in the Empower Health program. We applied human‐centered design methodology when developing the model to ensure the application was locally and culturally appropriate.^1^, ^2^ Of course, the model does not discourage traditional medicine or other resources that could potentially further patient education and improve adherence behaviors. However, traditional medicine practice is often not standardized and does not adhere to nationally accepted guidelines for cardiovascular care. For example, about 21% of South African traditional herbal medicine patients used combinations of tea and other mixtures for the treatment of hypertension.^3^ Such herbal medicines might have unpredictable interactions with prescribed medications.

Availability and accessibility of essential drugs remain a challenge in low‐ and lower‐middle income countries and improving therapy access is an important role of the responsible health care system and governments.^4^ At least occasional problems obtaining prescribed medications were reported by 20% of hypertensive patients in a recent Ghanaian survey.^5^ Indeed, the supply of antihypertensive medications was temporarily interrupted during the pilot phase of the Empower Health program, although significant improvements in blood pressure were still achieved (Figure 1).^2^

**FIGURE 1:**
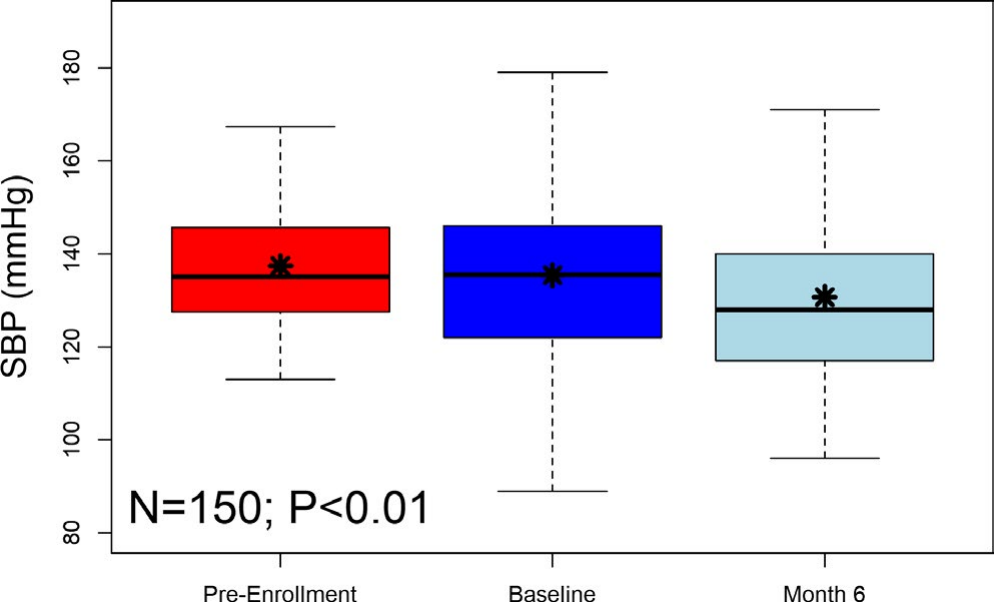
Empower Health model pilot study results. Systolic blood pressure was elevated prior to study, remaining elevated at baseline with a significant reduction through 6 months after enrollment in the program (−4.7 ± 18.7 mmHg, *p* < .01)^2^

While Kenya and Ghana are considered middle income countries, the population served by the model was mostly low‐income and middle‐income segments and in mostly remote rural settings. Both are representative of many Sub‐Saharan countries with rural and urban populations that face similar challenges controlling hypertension. Note that smart phone ownership is not required in the model. Shared access via trained personnel or community health workers or location‐based set‐ups accommodate all patients including most patients who do not own a smartphone. Furthermore, the Empower Health model is currently available to over 20 000 patients in 2 countries, serving both public and private sectors and growing. We intend to continue to report program outcomes and have planned new randomized multi‐center prospective studies. Based on this design and ongoing expansion, the Empower Health model is relevant to address low blood pressure control rates in many other African countries.

## CONFLICT OF INTEREST

FK, CL, and DH are full time employees of Medtronic Inc; RS has received consulting and speaker fees from Medtronic; all other authors report no competing interests.

## AUTHOR CONTRIBUTIONS

HAO, GY, CM, and DAH drafted the manuscript all authors reviewed, edited, and contributed materially to the final document.

## References

[1] Otieno HA , Miezah C , Yonga G , et al. Improved blood pressure control via a novel chronic disease management model of care in sub‐Saharan Africa: Real‐world program implementation results. J Clin Hypertens. 2021. 10.1111/jch.14174 PMC8678676 33471442

[2] Owusu IK , Adomako‐Boateng F , Kueffer F , et al. Novel hypertension management model of care improves blood pressure control in a West African Population. J Hypertens: Open Access. 2018;07(03). 10.4172/2167-1095.1000257

[3] Hughes GD , Aboyade OM , Clark BL , Puoane TR . The prevalence of traditional herbal medicine use among hypertensives living in South African communities. BMC Complement Altern Med. 2013;13:38. 23414344 10.1186/1472-6882-13-38 PMC3598715

[4] Husain MJ , Datta BK , Kostova D , et al. Access to cardiovascular disease and hypertension medicines in developing countries: an analysis of essential medicine lists, price, availability, and affordability. J Am Heart Assoc. 2020;9:e015302. 32338557 10.1161/JAHA.119.015302 PMC7428558

[5] Sarfo FS , Mobula LM , Burnham G , et al. Factors associated with uncontrolled blood pressure among Ghanaians: evidence from a multicenter hospital‐based study. PLoS One. 2018;13:e0193494. 29554106 10.1371/journal.pone.0193494 PMC5858765

